# Enhancing Dental Cement Bond Strength with Autofocus-Laser-Cutter-Generated Grooves on Polyetheretherketone Surfaces

**DOI:** 10.3390/polym15183670

**Published:** 2023-09-06

**Authors:** Tzu-Yu Peng, Tien-Li Ma, I-Ta Lee, Sheng-Han Wu, Yuichi Mine, Chia-Cheng Lin

**Affiliations:** 1 School of Dentistry, College of Oral Medicine, Taipei Medical University, Taipei 11031, Taiwan; typeng@tmu.edu.tw (T.-Y.P.); m204112006@tmu.edu.tw (S.-H.W.); 2 Research Center of Digital Oral Science and Technology, College of Oral Medicine, Taipei Medical University, Taipei 11031, Taiwan; 3 Department of Medical Systems Engineering, Graduate School of Biomedical and Health Sciences, Hiroshima University, Hiroshima 734-8553, Japan; mine@hiroshima-u.ac.jp; 4 Department of Dentistry, Shin Kong Wu Ho-Su Memorial Hospital, Taipei 11101, Taiwan; 5 School of Dental Technology, College of Oral Medicine, Taipei Medical University, Taipei 11031, Taiwan

**Keywords:** polyetheretherketone, surface treatment, autofocus laser, bond strength, CAD-CAM, resin cement

## Abstract

Polyetheretherketone (PEEK) is widely used in dentistry owing to its exceptional properties, including its natural appearance; however, existing surface treatment methods for bonding PEEK have limitations. Autofocus laser cutters, known for their precise engraving and cutting capabilities, offer potential for surface treatment of PEEK; thus, the objective of this study was to investigate the creation of laser groove structures on PEEK to enhance its bonding capability with dental resin cement. A dental computer-aided design and manufacturing system was used to fabricate PEEK samples, and three groove patterns (circle, line, and grid) were generated on PEEK surfaces, with air-abrasion used as the control group. The surface characteristics, cell viability, and bond strength were evaluated, and the data were statistically analyzed using one-way analysis of variance and post hoc Tukey’s tests (α = 0.05). Laser-treated PEEK exhibited a uniform texture with a groove depth of approximately 39.4 µm, hydrophobic properties with a contact angle exceeding 90°, a surface roughness of 7.3–12.4 µm, consistent topography, and comparable cell viability compared with untreated PEEK. Despite a decrease in bond strength after thermal cycling, no significant intergroup differences were observed, except for the line-shaped laser pattern. These findings indicate that the autofocus laser cutter effectively enhances the surface characteristics of PEEK by creating a uniform texture and grooves, showing promise in improving bonding properties, even considering the impact of thermal cycling effects.

## 1. Introduction

Polyetheretherketone (PEEK) is a semi-crystalline high-performance thermoplastic polymer material formed by connecting a phenylene ring backbone through ether and carbonyl bonds [1,2]. The stable structure of the phenyl rings renders PEEK molecules stiff, while providing exceptional dimensional, thermal, and chemical stability [3,4]. PEEK’s development began in aerospace and defense, then moved to industry [5,6]. By the late 1990s, it gained prominence in biomedicine due to its radiolucent nature, becoming a favored choice over metal implants in surgeries and orthopedics [7]. Subsequently, PEEK started being used in dentistry and was initially processed through casting techniques in granular or tablet forms. With the flourishing of digital dentistry, PEEK materials were developed into disk-shaped blocks suitable for dental computer-aided design and manufacturing (CAD-CAM) systems, such as VESTAKEEP (Polyplastics-Evonik Corporation Ltd. Japan) [8]. In addition to its mentioned physicochemical stability, PEEK’s low density of 1.4 g/cm^3^ is notable in dentistry for offering comfort and minimizing foreign body sensation in prosthetics [9]. PEEK’s elastic modulus (4–7 GPa), resembling that of the human mandible or teeth, allows for strong shock absorption and pressure relief, rendering it well-suited for dental implants or abutments, functioning as effective shock absorbers [10]. Previous research suggests PEEK’s strong biocompatibility with oral fibroblast cells, minimal inflammation, and positive osseointegration, potentially even impacting biofilm and reducing inflammation around implants [11,12,13]. Moreover, the easy reproducibility of PEEK using digital fabrication techniques further enhances its appeal in the field of dentistry [14,15]. The convergence of these factors has increasingly highlighted PEEK’s role and made it a favored material in dental applications [16].

To ensure a secure and strong bond with other dental materials, including resin cement, acrylic resin, and composite resin [17,18], PEEK surfaces require adequate pretreatment, typically mechanical and chemical pretreatments, before bonding [19]. Mechanical pretreatment typically involves air-abrasion using alumina oxide particles [20,21], with optimal parameters being a blasting pressure of 2 bar and an alumina particle size of 110 μm [22]. Chemical pretreatment often involves applying a primer containing methyl methacrylate (MMA) to the surface of PEEK, effectively enhancing its bonding properties [23,24]. Concentrated acid etching is mainly accomplished using a piranha solution and sulfuric acid. Research indicates that sulfuric acid etching effectively creates intricate surface pore structures and enhances bond strength [25,26], yet some studies, like Stawarczyk et al.’s research, have shown that solely using sulfuric acid to etch PEEK can increase surface roughness and surface energy, but it does not significantly enhance PEEK’s bonding strength [27]. Piranha solution, comprising sulfuric acid and hydrogen peroxide, acts as a strong oxidizing agent to remove organic residues from surfaces. Theoretically, it can enhance surface energy and functional groups of PEEK, promoting potential chemical bonding with other materials; however, Keul et al.’s research contradicts this, indicating that piranha solution does not effectively improve bonding with composite resins [28]. Nonetheless, the use of concentrated acid etching presents safety challenges due to the highly toxic nature of the acid, particularly in dental technician operations.

Many researchers have used lasers, including CO_2_ [29], neodymium-doped yttrium aluminum garnet (Nd:YAG) [30,31], erbium-doped yttrium aluminum garnet (Er:YAG) [32], erbium, chromium:yttrium-scandium-gallium-garnet [33], and potassium titanyl phosphate (KTP) [34], to irradiate dental materials, resulting in improved bond strength. However, the widespread adoption of computer numerical control (CNC) lasers in dental clinical practice is hindered by limitations such as device size and laser source intensity. Therefore, exploring alternative effective methods for surface treatment of PEEK to attain desirable bonding effects is essential for expanding its clinical utility. Autofocus laser cutters (ALCs) have found wide applications in material engraving, etching, and cutting, particularly in the production of leather and metal jewelry, enabling precise and intricate effects [35,36]. ALCs operate through computer software for design and control. They autofocus the laser beam energy onto the material surface according to the design file, enabling precise engraving and etching [37,38]. While air-abrasion has gained significant clinical popularity, the machinery associated with the blasting process might not always align with the specific requirements of typical dental clinics. In situations where dental equipment requires repair, executing surface treatments poses challenges and often involves the inconvenience of dispatching items to dental laboratories. In contrast, ALCs offer distinct advantages. They are compact, are easy to use, operate solely on power, and can be integrated with CAD-CAM systems, allowing for customizable patterns and structures. They exhibit remarkable versatility, enhancing their suitability for clinical settings. This underscores a notable shift, as ALCs not only simplify operational demands but also amplify their practicality within the realm of dental practice.

The objective of this study was to use an ALC to create groove structures with various morphologies on the surface of PEEK, with the aim of enhancing the bond strength between PEEK and dental resin cement. The hypothesis tested was that the laser-created groove structures would not yield a significant enhancement in the overall bonding efficacy of PEEK.

## 2. Materials and Methods

### 2.1. Sample Preparation and Surface Pretreatment

**Figure 1:**
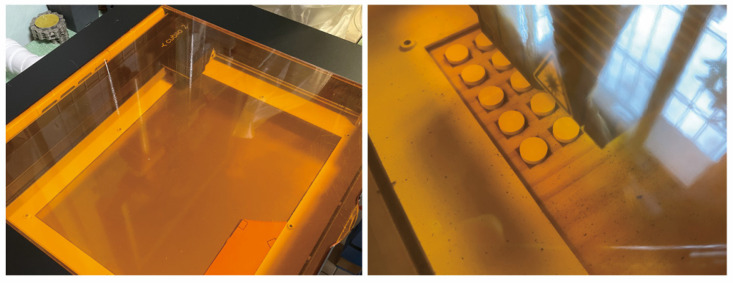
Autofocus laser cutter used in the current study.

All the materials used in this study are listed in Table 1. Experimental PEEK samples (Polyplastics-Evonik Corporation Ltd., Tokyo, Japan) with a diameter and thickness of 10 mm and 2.5 mm, respectively, were designed and fabricated using a dental CAD-CAM system (DGSHAPE DWX-52DCI; Roland DG Corp., Hamamatsu, Japan). A total of 146 pieces of PEEK samples were produced. The samples were cleaned with isopropanol, followed by steam, and then air-dried. Subsequently, an ALC (cubiio 2; MUHERZ Limited, Taiwan Branch (BVI), Taipei, Taiwan) was used to create groove structures on the PEEK surface (Figure 1). The structures were divided into three patterns: CP group, circle patterns; LP group, line patterns; and GP group, grid pattern. The ALC operated at a power of 20 W and speed of 8 mm/s, with five laser engraving passes performed on a specific area. As a control group (NP group), aluminum oxide sand (Cobra; Renfert GmbH, Hilzingen, Germany) was used for air-abrasion, with a blasting pressure of 0.2 MPa, particle size of 110 μm, sandblasting time of 10 s, and distance of 10 mm.

### 2.2. Cell Culture and Cell Metabolic Activity: Biocompatibility Evaluation

The human gingival fibroblast-1 (HGF-1) cell line was obtained from ATCC (#CRL-2014) and cultured in Dulbecco’s Modified Eagle Medium (DMEM) high glucose medium supplemented with 10% fetal bovine serum, 100 g/mL streptomycin, and 100 U/mL penicillin at 37 °C and 5% CO_2_. The medium was changed every three days. Cells at confluence from passages 3–7 were used for subsequent experiments. Autoclaved specimens (121 °C, 1.2 kg/cm^2^, 30 min) were transferred to a 24-well plate, and the HGF-1 cell line was seeded on top of each PEEK sample at a density of 3 × 10^6^ cells/well. While in direct contact, the HGF-1 cell line and PEEK samples (*n* = 3, *N* = 36) were incubated for 1, 2, and 4 days, and cell metabolic activity was subsequently assessed using PrestoBlue cell viability reagent (Invitrogen, Carlsbad, CA, USA) according to the manufacturer’s protocol.

### 2.3. Surface Characterization Analysis

The surface morphology of the samples was observed using a dental microscope at a magnification of 10× and an optical microscope (BA210; Motic Medical Diagnostic Systems, Co., Ltd., Xiamen, China) at magnifications of 40× and 100×. Surface patterns were observed and dimensions analyzed using a 3D optical microscope (VHX-700, Keyence Taiwan, Co., Ltd., Taipei, Taiwan) at a magnification of 500×. A contact angle analyzer (Phoenix Mini, Surface Electro Optics Co., Ltd., Gyeonggi-do, Republic of Korea) was used to determine the surface wettability and surface free energy (SFE) of the treated PEEK surfaces (*n* = 5 per group, *N* = 15). PEEK surface roughness (Ra) (*n* = 5 per group, *N* = 15) was assessed within a 50 × 50 mm area using a stylus profilometer (DektakXT, Bruker Taiwan Co., Ltd., Zhubei, Taiwan).

### 2.4. Bonding Strength Evaluation

A circular acrylic mold with an inner diameter of 5 mm was placed in the center of the pretreated PEEK samples to establish the bonding area. Resin cement (G-CEM LinkForce; GC Corp., Japan) was flowed into the mold, and a bonding cap was placed on top, applying a pressure of 4.9 N. Preliminary light curing was performed for 3 s, followed by the removal of excess resin cement and final light curing for 10 s. After completion of the bonding process, the samples were maintained in a chamber at 37 °C for 60 min. The samples were then divided into two groups (*n* = 10 per group, *N* = 80): one group was immersed in distilled water at 37 °C for 24 h, and the other group underwent 5000 cycles of artificial aging at 5–55 °C (ISO 10477 [39]). Shear bond strength (SBS) between the PEEK and resin cement was tested using a universal material testing machine (JSV-H1000; Algol Instrument Co., Ltd., Taoyuan, Taiwan), with shear force applied until fracture occurred. The mean and standard deviation of all SBS values were calculated. Following bond strength analysis, debonded surfaces were observed using a dental microscope at a magnification of 8× to determine the failure mode, categorized as adhesive failure (A failure), cohesive failure (C failure), or a combination of adhesive and cohesive failures (AC failure).

### 2.5. Statistical Analysis

The sample size for each group was calculated based on a G*Power software (version 3.1.9.6) with 80% power and 0.05 level of significance, which enabled clinically justified recommendations. Statistical software (SPSS, version 19, IBM Corporation, USA) was utilized to compute the mean and standard deviation for the entire dataset. Prior to further analysis, the normal distribution and variance homogeneity of all values underwent assessment through the Shapiro–Wilk test and Levene’s test, respectively. As the data in this study displayed a normal distribution, parametric analysis methods were employed. The biocompatibility evaluation experiments were replicated thrice for each specific assay. To analyze the data, we employed one-way analysis of variance (ANOVA). The comparisons among the results of surface wettability, SFE, and Ra values were performed using one-way ANOVA. Additionally, the SBS of PEEK and dental resin cement, after being subjected to various groove structures and thermal cycling, was assessed using two-way ANOVA. All comparisons among the multiple groups (CP, LP, GP, and NP) were examined through post hoc Tukey’s honest significant difference test. The significance level for all analyses was set at 5%.

## 3. Results

### 3.1. Surface Characteristics: Biological and Physical Properties

**Figure 2:**
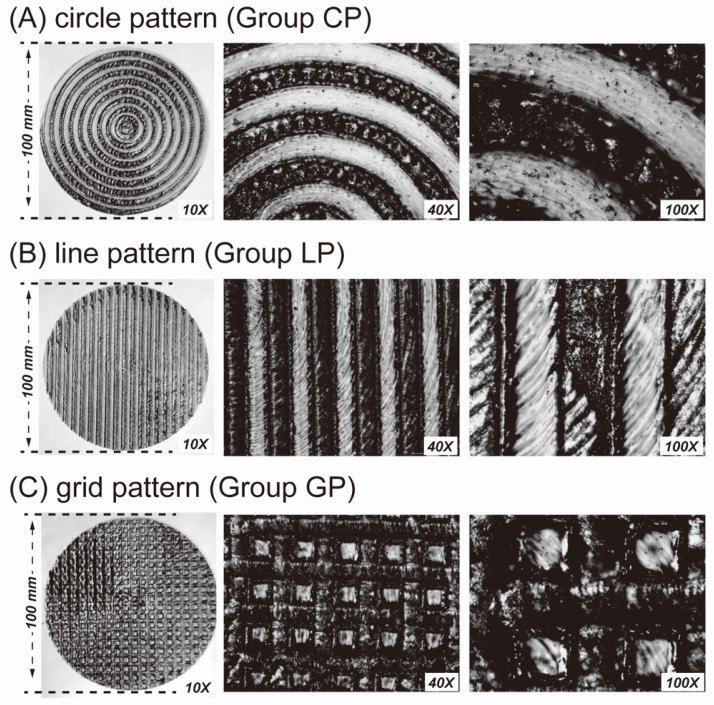
In each row, leftmost images show the macroscopic surface structure of the specimens captured using a 10× fixed-focus lens, and the middle and right images depict the submicroscopic surface structures of the specimens captured using 40× and 100× optical microscopes, respectively.

**Figure 3:**
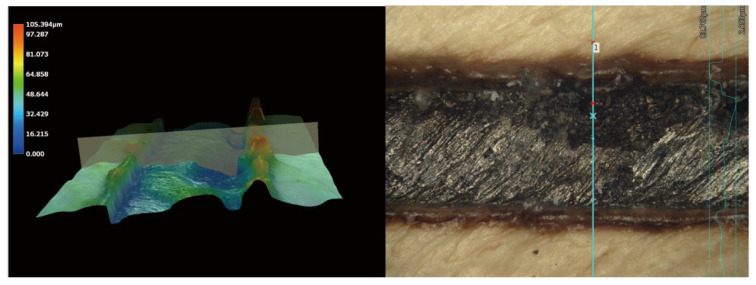
Observations made under a 500× 3D optical microscope to analyze surface patterns.

Microscope observations revealed a uniform texture on the PEEK surface, with dark areas corresponding to laser-created grooves, and lighter regions representing the PEEK materials (Figure 2). Further analysis using a 3D optical microscope revealed that the depth of the laser-created groove was approximately 39.45 µm (Figure 3). In group GP, the laser-induced grooves exhibited a melting phenomenon caused by the dense mesh-like structure, leading to reduced exposure of the PEEK material (Figure 2).

**Figure 4:**
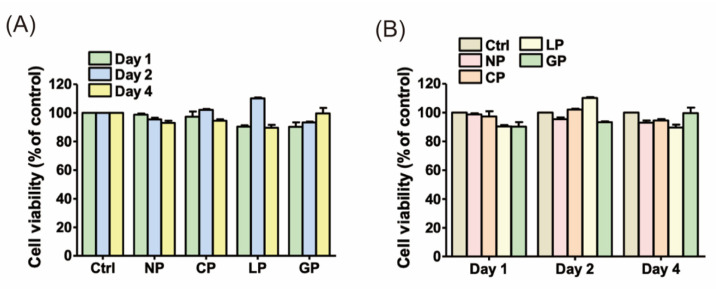
Cell metabolic activity of the HGF-1 cell line in direct contact with polyetheretherketone (PEEK) sample surfaces for 1, 2, and 4 days, assessed using PrestoBlue cell viability reagent. (**A**) Differences in the effects among different patterns. (**B**) Effects on the same patterns shown in (**A**) over specific time periods.

**Figure 5:**
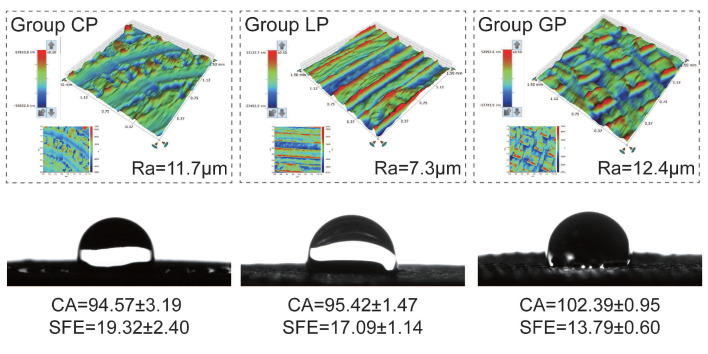
Surface roughness (Ra) measured using a stylus profilometer, and contact angle (CA) and surface free energy (SFE) determined using a contact angle analyzer. CA results are reported in degrees, whereas SFE results are expressed in mN/m.

Figure 4 illustrates the cell metabolic activity of the HGF-1 cell line in direct contact with PEEK material. The analysis results revealed that laser surface-treated PEEK showed comparable cell metabolic activity to untreated PEEK (*p* > 0.05). Furthermore, both treated and untreated PEEK samples did not significantly differ (*p* > 0.05) in cell metabolic activity compared with the blank control containing only DMEM. The samples exhibited a surface roughness ranging from 7.3 to 12.4 µm, and their surface topography remained consistent (Figure 5). Regarding surface wettability, all laser-treated samples demonstrated hydrophobic properties, with contact angles exceeding 90°. Specifically, group GP exhibited contact angles exceeding 100° (Figure 5), and the SFE ranged from 13.8 to 19.3 mN/m.

### 3.2. Bonding Performance: Bond Strength and Durability

**Figure 6:**
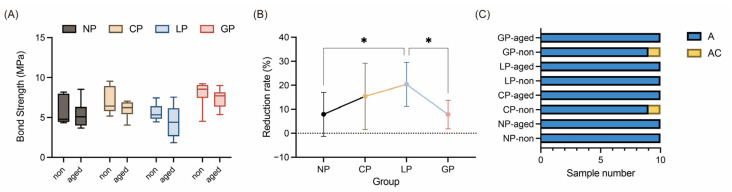
Results of (**A**) shear bond strength (SBS) tests, (**B**) the reduction rate of SBS between the aging and nonaging group (* means significant difference with other group, *p* < 0.05), and (**C**) the failure mode of the debonded surface. “Non” indicates specimens without thermal aging, whereas “aged” indicates specimens that underwent 5000 cycles of thermal aging. The labels “A” and “AC” correspond to adhesive failure and a mixed failure involving both adhesive and cohesive components, respectively.

Figure 6 shows the results of the SBS test and failure mode analysis. Prior to the artificial aging test, all samples exceeded the ISO 10477 specification [39] of a 5 MPa bond strength (Figure 6A). Among the tested groups, the GP group exhibited the highest SBS (8.01 MPa), followed by the CP (7.09 MPa), NP (5.73 MPa), and LP (5.62 MPa) groups. However, after undergoing the artificial aging test, SBS decreased for all samples (Figure 6B). The LP group exhibited the highest reduction rate (20.36%), followed by the CP (15.38%), NP (7.91%), and GP (7.80%) groups. Nevertheless, no significant differences were found among the groups (*p* > 0.473), although only the LP group fell below the 5 MPa standard (ISO 10477 [39]). In a comparison among groups, prior to artificial aging, the GP group exhibited a significantly higher SBS compared with the NP and CP groups (*p* < 0.05), and the CP group showed significantly higher SBS compared with the LP group (*p* < 0.05). After artificial aging, only the GP and NP groups differed significantly in terms of SBS (*p* < 0.05). Figure 6C presents the results of the debonded fracture surfaces after SBS testing, where the A failure mode was the predominant failure type, and only the CP and GP groups, in the nonaged cycle groups, had one sample with an AC failure.

## 4. Discussion

Laser engraving technology uses high-energy laser beams for noncontact surface engraving of materials [40,41]. The ALC is equipped with sensors and a control system that monitors the laser beam’s focal point position and automatically adjusts the cutting head’s height [37,38], optimizing cutting quality, reducing costs, and enhancing production efficiency and consistency [40,42]. Based on the present results, the ALC is proposed as a novel tool for surface treatment of PEEK to improve its properties and enhance bonding with resin cement. Existing literature confirms PEEK’s excellent biocompatibility [43,44], raising concerns about potential impacts on biocompatibility after laser treatment. However, direct culturing of laser-treated PEEK samples with the HGF-1 cell line resulted in no significant differences compared with untreated or blank samples, suggesting that ALCs do not compromise PEEK’s biocompatibility (Figure 4). Regarding the potential cytotoxicity of ALP, although it was not examined in our experiment, it is important to highlight the study conducted by Peng et al. [12]. They conducted a cytotoxicity comparison among various materials, including the same PEEK (VESTAKEEP) we utilized, as well as zirconia and titanium, using primary human oral fibroblasts (HOFs). Their findings revealed no statistically significant difference in cytotoxicity activity among HOFs exposed to different extracts, implying that VESTAKEEP is non-cytotoxic. With comparable outcomes in cellular activity and cytotoxicity, our experiment suggests that ALP treatment does not influence cellular activity, thereby implying that ALP is unlikely to cause cytotoxic effects.

Researchers have used CNC lasers (e.g., Nd:YAG, Er:YAG, and KTP) for pretreating restorative materials [29,30,31,32,34], significantly improving bonding performance by modifying the surface characteristics. However, these CNC lasers have high laser density and intensity, and their bulky nature limits convenience and practicality in dental practice. The compact size and lower emission intensities of ALCs make them a promising device for dental clinical practice [45]. Surface characteristics analysis (Figure 5) of ALC-treated PEEK samples revealed laser grooves with Ra values of 7.3–12.4 μm, comparable to Ra values obtained by Tsuka et al. [30] and Shabib et al. [31] (approximately 15.3 μm) after Nd:YVO_4_ laser treatment. Furthermore, Kimura et al. [46] reported an increase in the contact angle of PEEK surfaces from 116.1° to 126.5° following Nd:YVO_4_ laser treatment, aligning with the hydrophobic changes observed in the current study. Notably, ALC exhibits a similar capacity to modify material surface characteristics as Nd:YVO_4_ laser or other CNC laser systems.

The current study used ALC for PEEK surface treatment, resulting in improved bond strength between PEEK and resin, effectively refuting the null hypothesis. Tsuka et al. [30] and Shabib et al. [31] reported a SBS range of 13.2–16.3 MPa for PEEK surfaces treated with an Nd:YVO_4_ laser, and Ulgey et al. [34] achieved SBS values of 16.4 MPa and 11.3 Mpa when treating PEEK surfaces with Nd:YAG and KTP lasers, combined with a nanohybrid composite, respectively. In the current study, ALC-treated PEEK surfaces exhibited an SBS with resin cement ranging from 5.62 to 8.01 MPa before artificial aging (Figure 6). Although these SBS values were lower than those achieved with CNC lasers, they still exceeded the clinical threshold of 5 MPa specified by ISO 10477 [39]. Notably, the exclusion of primer smearing might have contributed to the lower SBS values in this study. MMA-based primers, such as visio.link (bredent, Senden, Germany) or HC primer (Shofu, Kyoto, Japan), have been reported in the literature to enhance bond strength between inert surfaces of high-performance polymer-based materials [18,23]. Incorporating a discussion on primer smearing in future considerations could lead to higher SBS values.

Airborne particle abrasion is widely used to treat various materials, including titanium alloys, zirconia ceramics, and thermoplastic high-performance polymers. In previous studies, the influence of different sandblasting conditions, such as particle size and blasting pressure, on the SBS of zirconia with composite resin [47], as well as PEEK with resin cement [22], was explored. Drawing on insights gained in the PEEK investigation [22], optimal conditions for airborne particle abrasion of PEEK in clinical applications involved using 110 μm alumina particles at a jet pressure of 0.2 MPa. PEEK samples treated under these conditions initially exhibited an SBS of 7.43 MPa, which decreased slightly to 6.68 MPa after artificial aging. These results suggest that this specific condition offers a satisfactory balance between bond strength and durability, preserving the integrity of PEEK without causing damage. Although the LP group fell below the desired threshold of 5 MPa (ISO 10477 [39]), both the CP and GP groups exhibited SBS values comparable to those achieved with air-abrasion and showed resistance to artificial aging. Therefore, ALCs can be considered a viable alternative surface treatment method, with the added advantage of automated operation, reducing human-induced variables and enhancing precision and homogeneity, leading to minimized errors, time-saving, and cost reduction.

Comparing the SBS of the CP group (with concentric circle patterns) and GP group (with grid patterns), the former exhibited slightly lower values. Specifically, the CP group showed an SBS of 7.09 MPa and 6.00 MPa for the nonaged and aged groups, respectively, whereas the GP group exhibited an SBS of 8.01 MPa and 7.36 MPa for the nonaged and aged groups, respectively. However, the statistical analysis revealed no significant difference between the two groups (nonaged group, *p* = 0.541; aged group, *p* = 0.209). From a clinical perspective, the concentric circle pattern may offer advantages owing to its alignment with the tooth axis, providing enhanced resistance against occlusal forces and mastication. Considering both experimental data and the clinical perspective, creating concentric circle patterns through laser engraving might represent the optimal design structure.

The current study possesses certain limitations. Firstly, it is crucial to note that the impact of ALC on PEEK could potentially lead to irreversible carbonization, thus compromising its visual appeal and aesthetic appearance. Secondly, a compelling need exists for enhanced precision in ALC equipment to enhance its effectiveness. Moreover, the evolving landscape of material surface treatments encompasses multifaceted approaches, including combining physical ALC with chemical primer applications. Notably, bonding performance is intricately linked to factors like the types of resin cement used. In this specific study, only one type of resin cement was employed (G-CEM Link Force, GC), potentially failing to comprehensively represent the entire spectrum of available resin cements in contemporary clinical dentistry. The aforementioned limitations and issues require further exploration and refinement in future research endeavors. This step is vital in facilitating the broader adoption of ALC within dental clinical practice.

## 5. Conclusions

The ALC was shown to be an impressive option for surface treatment, as evidenced through the remarkable uniform texture and intricate grooves it creates. These features contribute to its effectiveness in enhancing the surface characteristics of PEEK. Laser-treated PEEK exhibits hydrophobic properties, consistent surface topography, and comparable cell viability to that of untreated PEEK, further affirming the effectiveness of the ALC approach. Although the bond strength of all samples was affected by artificial aging, intriguing dynamics between different treatment groups emerged from intergroup comparisons. Overall, this study emphasizes the potential of laser surface treatment to enhance PEEK’s bond with resin cement, thus advancing its applicability in dentistry. Nevertheless, it is important to note that the irreversible carbonization caused by the laser process might affect the material’s appearance, which highlights the need for meticulous evaluation of aesthetic considerations in dental clinical scenarios.

## Figures and Tables

**Table 1:** Materials used in the present study.

Product Name	Composition	Manufacturer	Lot Number
VESTAKEEP (DC4450)	80% PEEK with 20% filler, including titanium dioxide and 1% pigment	Polyplastics-Evonik Corporation Ltd., Tokyo, Japan	57781699
G-CEM LinkForce	Paste A: bis-GMA, UDMA, dimethacrylate, etc. Paste B: bis-MEPP, UDMA, dimethacrylate, etc.	GC Corp., Tokyo, Japan	022009
Cobra (110 μm)	Al_2_O_3_, SiO_2_	Renfert GmbH, Hilzingen, Germany	2327409

## Data Availability

Not applicable.
